# HIV-1 neutralizing antibodies display dual specificity for the primary and coreceptor binding sites and preferential recognition of fully-cleaved Env

**DOI:** 10.1186/1742-4690-9-S2-P99

**Published:** 2012-09-13

**Authors:** Y Li, S O’Dell, R Wilson, X Wu, SD Schmidt, C Hogerkorp, MK Louder, N Longo, C Poulsen, J Guenaga, B Chakrabarti, N Doria-Rose, M Roederer, M Connors, JR Mascola, RT Wyatt

**Affiliations:** 1 IAVI/TSRI, LA Jolla, CA, USA; 2 VRC/NIAID/NIH, Bethesda, MD, USA; 3 NIAID/NIH, USA

## Background

The gp120 CD4 binding site (CD4bs) and coreceptor binding site (CoRbs) are two functionally conserved elements of the HIV-1 envelope glycoproteins (Env). We previously defined the presence of CD4bs neutralizing antibodies (nAbs) in the serum of an HIV-1 infected individual and subsequently isolated the CD4bs-specific monoclonal antibodies (mAbs) VRC01 and VRC03 from the memory B cell population. From the same patient sera, we detected that there was also a fraction of potent nAbs specific for elements of the Env CoRbs by differential protein adsorption followed by neutralization analysis.

## Methods

In this study, we employed a differential FACS-based sorting strategy using a stabilized gp120 core and a mutant gp140 possessing a CoRbs “knockout” mutation (I420R) to isolate CoRbs specific B cells.

## Results

The mAb VRC06 was recovered from these cells and its genetic sequence allowed us to identify a clonal relative, VRC06b, which had been isolated from a prior cell sort using a resurfaced core gp120 probe. VRC06 and VRC06b neutralized 22% and 44% of circulating viruses tested, respectively. Virus neutralization assays revealed that VRC06/VRC06b better neutralized autologous viruses compared to VRC01 and VRC03. More potent autologous neutralization was associated with a 7 amino acid residues insertion in the framework of the VH gene-coding region. Epitope mapping studies demonstrated that the two mAbs were sensitive to mutations in both the gp120 CD4bs and the CoRbs, including the gp120 bridging sheet and the base of the third major variable region (V3). Interestingly, cell-surface binding assays demonstrated their preferential recognition of fully-cleaved Env trimers compared to un-cleaved trimers.

## Conclusion

VRC06 and VRC06b are novel neutralizing mAbs that bind to a region of gp120 that overlaps with both the primary and secondary HIV-1 receptor binding sites, preferentially recognize fully-cleaved Env, and complement the neutralizing capacity of other CD4bs bNAbs isolated from the same individual.

